# Anti-Tuberculosis Agitation

**Published:** 1907-03

**Authors:** 


### ANTI-TUBERCULOSIS AGITATION.

It is a frequent comment that much urging is necessary to arouse the public's interest in sanitary matters, but when aroused sanitary results are often speedily forthcoming. The movement against tuberculosis is an instance in point. From small beginnings it has gained in volume until now the movement toward suppression of the disease is well nigh general and the effect is already apparent in the diminished death rate from tuberculosis manifested in mortuary statistics.

Georgia has not been laggard in this matter. It was the first of the Southern States to undertake any systematic investigation of the status of the disease. A State commission and a committee from the Georgia Medical Association did arduous work for the cause, and though the results are not immediately apparent, it is perfectly fair to presume that to their labors is due a more accurate conception among the laymen of the disease, a sure foundation upon which to build for the future.

That the laity is aroused in the matter is evident from the attitude of the newspapers, their willingness to print anti-tuberculosis literature and to give editorial aid and sanction to the movement.

An Association is now in the process of organization in Atlanta which will have for its avowed purpose, the inauguration of a deliberate and determined fight against the disease, availing itself of every means, educational and sanitary, to assist in the furtherance of its object. This association is destined to expand much beyond the confines of this city and it is more than likely that the personnel of the local association, made up of prominent and philanthropic laymen, as well as physicians, will establish emulation and the crusade will broaden out to include all parts of the State.

That practical results will proceed from the work of this Association can scarcely be doubted and it may not be an altogether idle dream to conceive of the disease which claims so many lives in the States, discomfited and vanquished in the end.

